# Gel Electrophoresis of an Oil Drop

**DOI:** 10.3390/gels11070555

**Published:** 2025-07-18

**Authors:** Hiroyuki Ohshima

**Affiliations:** Research Institute for Science and Technology, Tokyo University of Science, 2641 Yamazaki, Noda 278-8510, Chiba, Japan; ohshima@rs.noda.tus.ac.jp

**Keywords:** gel electrophoresis, gel electrophoretic mobility, oil drop, liquid drop, polymer gel medium, Marangoni effect

## Abstract

We present a theoretical model for the electrophoresis of a weakly charged oil drop migrating through an uncharged polymer gel medium saturated with an aqueous electrolyte solution. The surface charge of the drop arises from the specific adsorption of ions onto its interface. Unlike solid particles, liquid drops exhibit internal fluidity and interfacial dynamics, leading to distinct electrokinetic behavior. In this study, the drop motion is driven by long-range hydrodynamic effects from the surrounding gel, which are treated using the Debye–Bueche–Brinkman continuum framework. A simplified version of the Baygents–Saville theory is adopted, assuming that no ions are present inside the drop and that the surface charge distribution results from linear ion adsorption. An approximate analytical expression is derived for the electrophoretic mobility of the drop under the condition of low zeta potential. Importantly, the derived expression explicitly includes the Marangoni effect, which arises from spatial variations in interfacial tension due to non-uniform ion adsorption. This model provides a physically consistent and mathematically tractable basis for understanding the electrophoretic transport of oil drops in soft porous media such as hydrogels, with potential applications in microfluidics, separation processes, and biomimetic systems. These results also show that the theory could be applied to more complicated or biologically important soft materials.

## 1. Introduction

The electrophoretic motion of particles in polymer gel matrices [1,2,3,4,5,6,7,8,9,10,11,12,13,14,15,16,17,18,19,20,21,22,23,24,25,26,27,28,29] exhibits markedly different characteristics compared to that in free-electrolyte solutions [30,31,32,33,34,35]. Within a gel network, a migrating colloidal particle experiences two main types of interactions with the polymer matrix: one is the short-range steric resistance arising from frictional contact with the polymer segments, and the other is a long-range hydrodynamic effect mediated by the fluid flow around the particle. In the case of dilute gels—where the particle size is significantly smaller than the typical pore size—the electrophoretic mobility is often described as a product of the mobility due to hydrodynamic effects and a separate factor reflecting steric interference.

Over the years, theoretical treatments of gel electrophoresis have been developed for a variety of particle types, including rigid spheres [1,3,4,5,6,7,8,9,12,13,15,16,17,18], biopolymers [2], soft particles [10,11,14,19,20], and liquid drops [21,22,23]. Some studies have further addressed time-dependent transient behaviors [24,25,26,27,28,29]. A common modeling framework in these studies is to represent the polymer gel as a porous medium filled with electrolyte solution, applying an effective medium theory based on the Debye–Bueche–Brinkman formulation [36,37]. In this approach, the polymer segments are idealized as a continuous distribution of frictional obstacles that resist fluid motion.

While these theoretical developments have deepened our understanding of particle transport in gels, the practical significance of gel electrophoresis is equally notable. Electrophoretic techniques in polymer gels have been widely used in biochemistry and molecular biology, especially for the separation and analysis of proteins, nucleic acids, and other biomolecules. More recently, soft gel matrices, such as hydrogels and microstructured polymer networks, have found applications in areas such as targeted drug delivery, diagnostic devices, and responsive materials. In such applications, understanding the electrokinetic behavior of not only rigid particles but also emulsions and oil drops is crucial for optimizing transport and separation performance.

The electrokinetics of liquid drops presents unique features not found in solid particles. Unlike rigid particles, drops exhibit internal fluid motion, which modifies the overall flow field and alters the electrophoretic response [38,39,40,41,42,43,44,45,46,47,48,49,50,51,52]. Furthermore, the surface properties of drops are often governed by dynamic interfacial phenomena, including ion adsorption and Marangoni effects, which can lead to spatially varying surface properties under external fields. These complexities necessitate theoretical models that go beyond classical assumptions such as uniform surface charge or potential.

In many earlier theoretical studies on drop electrophoresis, the drop surface has been treated as having either a uniform surface charge density or a uniform surface potential. While such assumptions simplify the analysis, they may not accurately reflect the actual conditions where ions can dynamically adsorb to and desorb from the drop surface. As a result, spatial variations in surface potential and charge density can arise due to local ionic concentrations and electrochemical gradients.

In the present study, we investigate the electrophoretic mobility of weakly charged oil drops in an uncharged dilute gel medium under an applied electric field. Our analysis builds upon a simplified version of the Baygents–Saville theory [40], which assumes that no mobile ions reside inside the drop and that the drop’s surface charge originates from the selective adsorption of ions on the drop surface. Importantly, in contrast to previous models, the present formulation allows for both the surface potential and surface charge density of the drop to vary along the drop surface in response to the applied electric field.

The electrophoretic motion of the drop is driven primarily by long-range hydrodynamic effects with the gel matrix, described using the Brinkman–Debye–Bueche framework [36,37], and is further influenced by Marangoni stresses arising from gradients in interfacial tension. These effects are incorporated into a consistent theoretical model that yields an analytical expression for the electrophoretic mobility of the drop.

In a previous study [52], we developed a theoretical model for the electrophoresis of oil drops in free solution, based on the Baygents–Saville framework [40]. In the present work, we extend that model to the case of the electrophoresis of oil drops in an uncharged polymer gel medium. The resulting formulation provides a theoretical basis for understanding and predicting the electrokinetic behavior of oil drops in soft porous environments such as hydrogels, which are increasingly important in microfluidic systems, bioanalytical devices, and soft-matter engineering.

## 2. Results and Discussion

### 2.1. Fundamental Electrokinetic Equations

**Figure 1:**
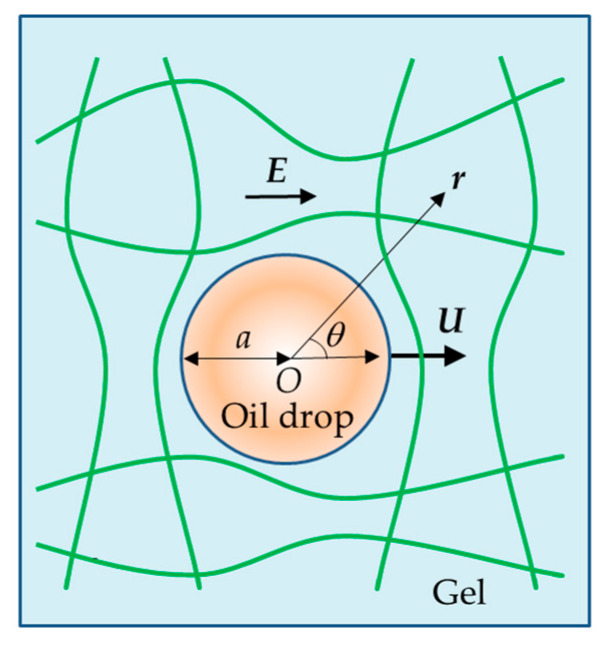
Electrophoresis of an oil drop with velocity ***U*** in an uncharged polymer gel medium under an applied electric field ***E***.

Consider a charged spherical oil drop of radius *a*, zeta potential *ζ*, and relative permittivity *ε*_d_ moving with a steady velocity ***U*** in an uncharged polymer gel medium of effectively infinite extent containing an electrolyte solution of viscosity *η* and relative permittivity *ε*_r_. The motion of the drop is driven by an applied electric field ***E*** (Figure 1).

The oil drop is modeled following the theory of Baygents and Saville [40], in which the drop surface is charged by ion adsorption. The polymer gel is treated as a porous continuum based on the Brinkman–Debye–Bueche continuum medium [36,37], where the polymer segments are idealized as uniformly distributed frictional centers that resist fluid flow within the gel. The electrolyte solution is composed of *N* ionic species, each with valence *z_i_*, bulk concentration (number density) $n_{i}^{\infty }$, and drag coefficient *λ_i_* (*i* = 1, 2, …, *N*). The electroneutrality condition of the system is $\sum_{i=1}^{N}z_{i}{en}_{i}^{\infty }=0$, where *e* is the elementary electric charge. Throughout this study, we adopt a reference frame that is fixed at the center of the spherical oil drop. Accordingly, the origin of the spherical coordinate system (*r*, *θ*, *ϕ*) is located at the drop’s center, and the polar axis (*θ* = 0) is taken to be parallel to the externally applied electric field ***E***. For a spherical drop, the electrophoretic velocity ***U*** is directed along ***E***. In the absence of the applied electric field ***E***, the drop surface at *r* = *a* is assumed to carry a uniform zeta potential *ζ*, where *r* = |***r***|, ***r*** being the position vector. The analysis is based on the following assumptions. (i) The Reynolds numbers for the fluid motion both inside and outside the drop are sufficiently small, allowing neglecting inertial terms in the Navier–Stokes equations; the fluids are considered incompressible. (ii) The applied electric field ***E*** is weak, so the resulting drop velocity ***U*** varies linearly with ***E***, and nonlinear effects due to stronger fields can be ignored. (iii) The interior of the drop contains no dissolved ions. (iv) Electrolyte ions exhibit linear adsorption behavior at the drop surface, where the surface density of adsorbed ions is proportional to the surrounding ion concentration, scaled by an adsorption coefficient.

We describe the fluid motion in a coordinate frame moving with the drop. The origin remains fixed at the drop’s center, and the drop velocity is given by ***U***(*U*cos*θ*, −*U*sin*θ*, 0), while the electric field is expressed as ***E***(*E*cos*θ*, −*E*sin*θ*, 0), where *U* and *E* denote the signed magnitude of the drop velocity ***U*** and the magnitude of the electric field ***E***, respectively.

The governing electrokinetic equations for the flow velocity ***u***(***r***) = (***u***_r_(r), *u_θ_*(r), 0) of the liquid outside the drop, the flow velocity inside the drop ***u***_I_(***r***) = (*u*_I*r*_(***r***), *u*_I_*_θ_*(***r***), 0), and for the velocity $v_{i}\left(r\right)=(v_{ir}\left(r\right),v_{i\theta }\left(r\right),0)$ of the i-th ionic species (*i* = 1, 2, …, *N*) are given as follows. Note that, owing to the rotational symmetry about the polar (*θ* = 0) axis, all relevant variables depend solely on the radial coordinate *r* and the polar angle *θ*.

(1) $$\eta \nabla \times \nabla \times u\left(r,\theta \right)+\nabla p\left(r,\theta \right)+\rho_{e}\left(r\right)\nabla \psi \left(r,\theta \right)+\gamma \left(u\left(r,\theta \right)+U\right)=0$$

(2) $$\eta \nabla \times \nabla \times u_{I}\left(r,\theta \right)+\nabla p_{I}\left(r,\theta \right)=0$$

(3) $$\nabla \cdot u\left(r,\theta \right)=0$$

(4) $$\nabla \cdot u_{I}\left(r,\theta \right)=0$$

(5) $$v_{i}\left(r,\theta \right)=u\left(r,\theta \right)-\frac{1}{\lambda_{i}}\nabla \mu_{i}\left(r,\theta \right)$$

(6) $$\mu_{i}\left(r,\theta \right)=\mu_{i0}+z_{i}e\psi \left(r,\theta \right)+kTlnn_{i}\left(r,\theta \right)$$

(7) $$\nabla \cdot \left(n_{i}\left(r,\theta \right)v_{i}\left(r,\theta \right)\right)=0$$

(8) $$∆\psi \left(r,\theta \right)=-\frac{\rho_{e}\left(r,\theta \right)}{\varepsilon_{r}\varepsilon_{0}}$$

(9) $$\rho_{e}\left(r,\theta \right)=\sum_{i=1}^{N}z_{i}en_{i}\left(r,\theta \right)$$

(10) $$∆\psi_{I}\left(r,\theta \right)=0$$

Here, *p*(*r*, *θ*) and *p*_I_(*r*, *θ*) denote the pressures outside and inside the drop, respectively. The space charge density *ρ*_e_(*r*, *θ*), arising from electrolyte ions, is defined by Equation (9). The electric potentials outside and inside the drop are represented by *ψ*(*r*, *θ*) and *ψ*_I_(*r*, *θ*), respectively. For the *i*-th ionic species at position ***r***, *μ_i_*(*r*, *θ*) and *n_i_*(*r*, *θ*) correspond to its electrochemical potential and concentration. The term *μ_i_*_0_ is a constant part of *μ_i_*(*r*, *θ*). Boltzmann’s constant is given by *k*, the absolute temperature by *T*, and *ε*_0_ denotes the vacuum permittivity. Here, the subscript capital I denotes quantities inside the oil drop, namely, the fluid velocity ***u***_I_(*r*, *θ*), pressure ***p***_I_(*r*, *θ*), and the electric potential *ψ*_I_(*r*, *θ*).

Equations (1)–(4) correspond to the Navier–Stokes equations and the continuity equation describing steady-state incompressible flow, under assumption (i), in the presence of body forces –*ρ*_e_∇*ψ*. The frictional force exerted on polymer segments within the gel medium by the liquid flow is represented by the term *γ*(***u***(***r***) + ***U***) in Equation (1), where *γ* is the friction coefficient as defined in the Brinkman–Debye–Bueche framework [36,37].

Equation (5) indicates that the flux ***v****_i_*(*r*, *θ*) of the *i*-th ionic species is driven by the liquid velocity ***u***(*r*, *θ*) and the spatial gradient of the electrochemical potential *μ_i_*(*r*, *θ*), as expressed in Equation (6). The continuity equation for the *i*-th ionic species in the electrolyte solution is given by Equation (7). Equation (8) represents the Poisson equation valid outside the drop (*r* > *a*), whereas Equation (10) is the Laplace equation applicable within the drop (0 ≤ *r* < *a*). 

The electrolyte ions are adsorbed onto the drop surface. We define $n_{i}^{s}\left(\theta \right)$, $\mu_{i}^{s}\left(\theta \right)$, $v_{i}^{s}\left(\theta \right)$, and $\lambda_{i}^{s}$ as the surface number density, electrochemical potential, velocity, and drag coefficient of the *i*-th adsorbed ionic species, respectively. These quantities are functions of the polar angle *θ* alone and are governed by the following set of equations:

(11) $$v_{i}^{s}\left(\theta \right)=u\left(a,\theta \right)-\frac{1}{\lambda_{i}^{s}}\nabla \mu_{i}^{s}\left(\theta \right)$$

(12) $$\mu_{i}^{s}\left(\theta \right)=\mu_{i0}^{s}+z_{i}e\psi \left(a,\theta \right)+kTlnn_{i}^{s}\left(\theta \right)$$

(13) $$\nabla_{s}\cdot \left(n_{i}^{s}\left(\theta \right)v_{i}^{s}\left(\theta \right)\right)+n_{i}\left(a,\theta \right)v_{ir}\left(a,\theta \right)=0$$

(14) $$n_{i}^{s}\left(\theta \right)=K_{i}n_{i}\left(a,\theta \right)$$

(15) $$\sigma \left(\theta \right)=\sum_{i=1}^{N}{z_{i}en}_{i}^{s}\left(\theta \right)=\sum_{i=1}^{N}{K_{i}z_{i}en}_{i}\left(a,\theta \right)$$

(16) $$\gamma \left(\theta \right)=\gamma_{0}-kT\sum_{i=1}^{N}n_{i}^{s}\left(\theta \right)=\gamma_{0}-kT\sum_{i=1}^{N}{K_{i}n}_{i}\left(a,\theta \right)$$

where $\mu_{i0}^{s}$ is constant independent of *r*. Equation (11) describes the surface flow $v_{i}^{s}\left(\theta \right)$ of the *i*-th ionic species adsorbed on the drop surface, which results from two contributing factors: the local fluid velocity at the drop surface, ***u***(*a*, *θ*), and the tangential gradient of the electrochemical potential $\mu_{i}^{s}\left(\theta \right)$, given by Equation (12). Equation (13) represents the surface continuity condition for the *i*-th ionic species, where ∇_s_ denotes the surface divergence operator. As expressed in Equation (14), adsorption of the *i*-th ionic species onto the drop surface is assumed to follow a linear isotherm as per assumption (iv), with adsorption strength characterized by the constant *K_i_*. This ion adsorption gives rise to a non-uniform surface charge distribution *σ*(*θ*), described by Equation (15). The constant *K_i_* is nonzero only for ions capable of adsorbing to the drop surface; it is set to zero for non-adsorbing species, such as those derived from the supporting electrolyte. The surface tension *γ*(*θ*) of the drop surface is given by Equation (16), where *γ*_0_ is the surface tension in the absence of the ion adsorption. The second term in Equation (16) quantifies the reduction in surface tension due to the linear adsorption of ions onto the drop surface.

The velocity fields of the liquid, ***u***(*r*, *θ*), must satisfy the following boundary conditions at the drop surface (*r* = *a*) and at a point far from the drop (*r* → ∞). At *r* = *a*, the flow velocities outside and inside the drop, ***u***(*r*,*θ*) and ***u***_I_(*r*,*θ*), coincide, and their normal components vanish at the drop surface, as given below.

(17) $$u_{r}\left(a,\theta \right)=u_{Ir}\left(a,\theta \right)=0$$

(18) $$u_{\theta }\left(a,\theta \right)=u_{I\theta }\left(a,\theta \right)$$

and

(19) $$u\left(r\right)\to -U\;as\;r\to \;\infty$$

The condition for the continuity of the tangential stress at the drop surface—accounting for both hydrodynamic and Maxwell stress components as well as the Marangoni effect—is given by [40]

(20) $$\left\{\sigma_{r\theta }^{H}\left(a,\theta \right)+\sigma_{r\theta }^{M}\left(a,\theta \right)\right\}-\left\{\sigma_{Ir\theta }^{H}\left(a,\theta \right)+\sigma_{Ir\theta }^{M}\left(a,\theta \right)\right\}+\frac{1}{a}\frac{d\gamma (\theta )}{d\theta }=0$$

Here $\sigma_{r\theta }^{H}(r,\theta )$ and $\sigma_{r\theta }^{M}(r,\theta )$, respectively, denote the tangential components of hydrodynamic and Maxwell stresses outside the drop, and $\sigma_{Ir\theta }^{H}(r,\theta )$ and $\sigma_{Ir\theta }^{M}(r,\theta )$ are the corresponding components inside the drop.

The electric potentials *ψ*(*r*, *θ*) and *ψ*_I_(*r*, *θ*) and the ionic concentration *n_i_*(*r*, *θ*) of the i-th ionic species must satisfy the following boundary conditions:

(21) $$\psi_{I}\left(a,\theta \right)=\psi \left(a,\theta \right)$$

(22) $${\left.\varepsilon_{d}\frac{\partial \psi_{I}\left(r,\theta \right)}{\partial r}\right|}_{r=a^{-}}-{\left.\varepsilon_{r}\frac{\partial \psi \left(r,\theta \right)}{\partial r}\right|}_{r=a^{+}}=\frac{\sigma \left(\theta \right)}{\varepsilon_{0}}=\frac{1}{\varepsilon_{0}}\sum_{i=1}^{N}{K_{i}z_{i}en}_{i}\left(a,\theta \right)$$

Since the disturbance in *ψ*(*r*, *θ*) and in *n_i_*(*r*, *θ*) due to the presence of the drop become negligible far from the drop, we have

(23) $$\psi \left(r,\theta \right)\to -Ercos\theta \;\;as\;r\;\to \infty$$

(24) $$n_{i}\left(r,\theta \right)\to n_{i}^{\infty }\;\;as\;r\;\to \infty$$

Additionally, in the stationary state, the net force acting on the drop must be zero.

Assuming that the equilibrium ionic concentration *n_i_*^(0)^(*r*) obeys the Boltmann distribution, the charge density *ρ*_e_^(0)^(*r*), and electric potential *ψ*^(0)^(*r*) satisfy

(25) $$n_{i}^{\left(0\right)}\left(r\right)=n_{i}^{\infty }exp\left(-\frac{z_{i}e\psi^{\left(0\right)}\left(r\right)}{kT}\right)$$

(26) $$∆\psi^{\left(0\right)}\left(r\right)=-\frac{\rho_{e}^{\left(0\right)}\left(r\right)}{\varepsilon_{r}\varepsilon_{0}}$$

(27) $$\rho_{e}^{\left(0\right)}\left(r\right)=\sum_{i=1}^{N}z_{i}en_{i}^{\left(0\right)}\left(r\right)=\sum_{i=1}^{N}z_{i}en_{i}^{\infty }exp\left(-\frac{z_{i}e\psi^{\left(0\right)}\left(r\right)}{kT}\right)$$

Combining Equations (25)–(27), we obtain the following Poisson–Boltzmann equations:

(28) $$∆\psi^{\left(0\right)}\left(r\right)=-\frac{1}{\varepsilon_{r}\varepsilon_{0}}\sum_{i=1}^{N}z_{i}en_{i}^{\infty }exp\left(-\frac{z_{i}e\psi^{\left(0\right)}\left(r\right)}{kT}\right)$$

The boundary conditions for *ψ*^(0)^(*r*) are $\psi^{\left(0\right)}\left(a\right)=\zeta$ and $\psi^{\left(0\right)}\left(r\right)\to 0\;as\;r\;\to \infty .$

The equilibrium number density of the *i*-th ionic species adsorbed onto the drop surface is denoted by $n_{i}^{s,(0)}$ and is given by

(29) $$n_{i}^{s,(0)}=K_{i}n_{i}^{\left(0\right)}\left(a\right)=K_{i}n_{i}^{\infty }exp\left(-\frac{z_{i}e\zeta }{kT}\right)$$

Using Equation (29), the equilibrium surface charge density denoted as *σ*^(0)^ is given by

(30) $$\sigma^{\left(0\right)}=\sum_{i=1}^{N}{z_{i}en}_{i}^{s,(0)}=\sum_{i=1}^{N}K_{i}z_{i}en_{i}^{\left(0\right)}\left(a\right)=\sum_{i=1}^{N}K_{i}z_{i}en_{i}^{\infty }exp\left(-\frac{z_{i}e\zeta }{kT}\right)$$

Here, $\psi_{I}^{\left(0\right)}$ and $n_{i}^{s,\left(0\right)}$ are constants that do not depend on *r*. This is because, in the absence of the applied electric field ***E***, there are no electrolyte ions within the drop, and the adsorbed ions are uniformly distributed over the drop surface. Under these conditions, the electric potential inside the drop, which is obtained as the solution to the Laplace equation (Equation (10)), remains constant throughout the interior. We thus obtain

(31) $${\left.\frac{d\psi^{\left(0\right)}(r)}{dr}\right|}_{r=a^{+}}=-\frac{\sigma^{\left(0\right)}}{\varepsilon_{r}\varepsilon_{0}}=-\frac{1}{\varepsilon_{r}\varepsilon_{0}}\sum_{i=1}^{N}K_{i}z_{i}en_{i}^{\infty }exp\left(-\frac{z_{i}e\zeta }{kT}\right)$$

For the low potential case, Equation (28) can be linearized to give

(32) $$∆\psi^{\left(0\right)}\left(r\right)=\kappa^{2}\psi^{\left(0\right)}\left(r\right)$$

where

(33) $$\kappa ={\left(\frac{\sum_{i=1}^{N}z_{i}^{2}e^{2}n_{i}^{\infty }}{\varepsilon_{r}\varepsilon_{0}kT}\right)}^{1/2}$$

is the Debye–Hückel parameter, and 1/*κ* is the Debye length. Solving Equation (32), we obtain

(34) $$\psi^{\left(0\right)}\left(r\right)=\zeta \frac{a}{r}e^{-\kappa (r-a)}$$

with

(35) $$\zeta =\frac{a\sigma^{\left(0\right)}}{\varepsilon_{r}\varepsilon_{0}(1+\kappa a)}=\frac{a}{\varepsilon_{r}\varepsilon_{0}(1+\kappa a)}\sum_{i=1}^{N}K_{i}z_{i}en_{i}^{\infty }$$

(36) $$\sigma^{\left(0\right)}=\sum_{i=1}^{N}K_{i}z_{i}en_{i}^{\infty }$$

Equations (34)–(36) represent the potential distribution *ψ*^(0)^(*r*) around a weakly charged oil drop, its zeta potential *ζ*, and equilibrium surface charged density *σ*^(0)^, respectively.

### 2.2. Weak-Field Approximation

For a weak field ***E***, the deviations of *n_i_*(*r*, *θ*), *ψ*(*r*, *θ*), *ψ*_I_(*r*, *θ*), *ρ*_e_(*r*, *θ*), *μi*(*r*,*θ*), $n_{i}^{s}\left(\theta \right)$, and $\mu_{i}^{s}\left(\theta \right)$ from their equilibrium values (i.e., those in the absence of the applied electric field ***E***) due to the applied field ***E*** are small. Under this condition, each of these quantities can be expressed as

(37) $$X\left(r,\theta \right)=X^{\left(0\right)}\left(r\right)+\delta X\left(r,\theta \right)$$

where $X\left(r,\theta \right)$ represents any of these quantities, and quantities with superscript (0) refer to those at equilibrium.

Symmetry considerations permit us to write the liquid velocity ***u***(*r*, *θ*) outside the drop and the deviation *δμ_i_*(*r*, *θ*) of the electrochemical potential *μ_i_*(*r*, *θ*) of the *i*-th ionic species as

(38) $$u\left(r\right)=\left(-\frac{2}{r}h\left(r\right)Ecos\theta ,\;\frac{1}{r}\frac{d}{dr}(rh\left(r\right))Esin\theta ,\;0\right)$$

(39) $$\delta \mu_{i}\left(r,\theta \right)=-z_{i}e\phi_{i}\left(r\right)Ecos\theta$$

where *h*(*r*) and *ϕi*(*r*) are functions of *r*. It can be shown that, as in the case of the electrophoresis of an ion-adsorbed oil drop [52], the fundamental electrokinetic equations (Equations (1)–(16)) reduce to the following two equations for *h*(*r*) and *ϕ_i_*(*r*):

(40) $$L(Lh-\lambda^{2}h)=G(r)$$

(41) $$L\phi_{i}=g_{i}(r)$$

with

(42) $$G\left(r\right)=-\frac{e}{\eta r}\frac{dy}{dr}\sum_{i=1}^{N}z_{i}^{2}n_{i}^{\infty }e^{-z_{i}y}\phi_{i}\left(r\right)$$

(43) $$g_{i}\left(r\right)=\frac{dy}{dr}\left(z_{i}\frac{d\phi_{i}}{dr}-\frac{2\lambda_{i}}{e}\frac{h}{r}\right)$$

(44) $$\lambda ={\left(\frac{\gamma }{\eta }\right)}^{1/2}$$

where *λ* is the reciprocal of the Brinkman screening length 1/*λ*,

(45) $$L=\frac{d}{dr}\frac{1}{r^{2}}\frac{d}{dr}r^{2}=\frac{d^{2}}{dr^{2}}+\frac{2}{r}\frac{d}{dr}-\frac{2}{r^{2}}$$

is a differential operator, and *y*(*r*) = *eψ*^(0)^(*r*)/*kT* is the scaled equilibrium electric potential.

The boundary conditions for *h*(*r*) and *ϕ_i_*(*r*) reduce to

(46) $$h(a^{+})=0$$

(47) $${\left.\frac{dh}{dr}\right|}_{r=a^{+}}-\frac{\eta a}{3\eta_{d}}{\left.\frac{d^{2}h}{dr^{2}}\right|}_{r=a^{+}}=-\frac{1}{3\eta_{d}}\sum_{i=1}^{N}{K_{i}z}_{i}en_{i}^{\infty }exp\left(-\frac{z_{i}e\zeta }{kT}\right)\phi_{i}\left(a\right)$$

(48) $${\left.\frac{d^{3}h}{dr^{3}}\right|}_{r=a^{+}}-\frac{5}{a}{\left.\frac{d^{2}h}{dr^{2}}\right|}_{r=a^{+}}=2\int_{a}^{\infty }\left(1-\frac{3r^{3}}{2a^{3}}\right)G\left(r\right)dr$$

(49) $$h\left(r\right)\to \frac{U}{2E}r\;\;as\;r\;\to \infty$$

(50) $${\left.\frac{d\phi_{i}(r)}{dr}\right|}_{r=a^{+}}+2K_{i}\left\{\frac{\lambda_{i}}{z_{i}ea}{\left.\frac{dh(r)}{dr}\right|}_{r=a^{+}}-\frac{\lambda_{i}}{\lambda_{i}^{s}}\frac{\phi_{i}(a)}{a^{2}}\right\}=0\;$$

(51) $$\phi_{i}(r)\to 0\;as\;r\;\to \infty \;\;\;$$

### 2.3. General Mobility Expression

It follows from Equation (49) that the gel electrophoretic mobility *μ* of an oil drop, which is defined by ***U*** = *μ**E***, is given by

(52) $$\mu =\frac{U}{E}=\underset{r\to \infty }{\lim}\frac{2h(r)}{r}$$

By solving Equation (40) with the boundary conditions given in Equations (46)−(48), and substituting the result into Equation (52), we obtain

$$\mu =\frac{2}{3\lambda^{2}\Omega }\int_{a}^{\infty }\left\{\left(1+\lambda r\right)e^{-\lambda \left(r-a\right)}-\left(1+\lambda a\right)-\frac{\lambda^{2}a^{2}}{3}\left(\frac{3\eta_{d}+3\eta +\eta \lambda a}{3\eta_{d}+2\eta }\right)\left(1-\frac{r^{3}}{a}\right)\right\}G(r)dr$$

(53) $$-\frac{2\left(1+\lambda a\right)}{3\Omega \left(3\eta_{d}+2\eta \right)}\;\sum_{i=1}^{N}K_{i}z_{i}en_{i}^{\infty }exp\left(-\frac{z_{i}e\zeta }{kT}\right)\phi_{i}\left(a\right)$$

with

(54) $$\Omega =1+\lambda a+\left(\frac{3\eta_{d}+3\eta +\eta \lambda a}{3\eta_{d}+2\eta }\right)\frac{\lambda^{2}a^{2}}{9}$$

where Ω represents the modification factor for the Stokes drag on an oil drop moving through a gel medium. 

Similarly, by solving Equation (41) with the boundary conditions given in Equations (50) and (51), we obtain

$$\phi_{i}\left(r\right)=r-\frac{r}{3}\int_{r}^{\infty }g_{i}\left(x\right)dx-\frac{1}{3r^{2}}\int_{a}^{r}r^{3}g_{i}\left(r\right)dr-\frac{a^{3}}{6r^{2}}\left(\frac{\lambda_{i}^{s}a-2K_{i}\lambda_{i}}{\lambda_{i}^{s}a+K_{i}\lambda_{i}}\right)\int_{a}^{\infty }g_{i}\left(r\right)dr$$

(55) $$+\frac{a^{3}}{2r^{2}}\left(\frac{\lambda_{i}^{s}a-2K_{i}\lambda_{i}}{\lambda_{i}^{s}a+K_{i}\lambda_{i}}\right)+\frac{a^{3}}{r^{2}}\left(\frac{K_{i}\lambda_{i}^{s}\lambda_{i}}{\lambda_{i}^{s}a+K_{i}\lambda_{i}}\right)\frac{1}{z_{i}e}{\left.\frac{dh}{dr}\right|}_{r=a^{+}}$$

By evaluating the value of *ϕ_i_*(*r*) at *r* = *a* using Equation (55), and substituting the resulting value *ϕ_i_*(*a*) into Equation (53), the gel electrophoretic mobility *μ* of the oil drop can be calculated. Note that Equations (50) and (55) correspond to Equations (94) and (97) in our previous paper [52], which contained errors: $\lambda_{i}^{s}$ should be replaced by $\lambda_{i}^{s}a$ and 2*r*^2^ in the last term of Equation (97) should be replaced by *r*^2^.

Equation (53) is the required general expression for the electrophoretic mobility *μ* of a spherical oil drop of radius a carrying zeta potential *ζ* in an uncharged polymer gel medium.

### 2.4. Numerical Results and Discussion

In this study, the stress balance at the surface of a weakly charged oil drop is formulated by taking into account not only the hydrodynamic stress but also the Maxwell stress and the Marangoni stress. When an oil drop moves in an external electric field, gradients of electric potential and ion concentration develop along its surface. In contrast, for a mercury drop [39], the continuity condition of the hydrodynamic stress alone suffices to describe the system. For the oil drop, in addition to the hydrodynamic stress, these gradients tend to induce additional stresses—namely, the Maxwell and Marangoni stresses—which act to reduce the gradients themselves. As a result, the motion of the oil drop is suppressed. This perspective provides an intuitive understanding of how the Marangoni effect and Maxwell stress contribute to decreasing the electrophoretic mobility of the drop.

We derived an approximate expression for the electrophoretic mobility, accurate to the first order in the zeta potential *ζ* (Equation (53)). Using this result, we obtain a simplified closed-form analytic formula for the electrophoretic mobility *μ* of a weakly charged oil drop in an uncharged polymer gel medium. In the regime of low zeta potential, the equilibrium potential distribution *ψ*^(0)^(*r*) outside the drop is given by Equation (34). Furthermore, we assume that the drag coefficient $\lambda_{i}^{s}$ of ions adsorbed on the drop surface is significantly larger than that of freely moving ions in the bulk, denoted by *λ_i_*. This assumption is justified in most practical cases, as adsorbed ions experience substantial resistance due to their restricted motion along the surface and interactions with the drop surface, unlike bulk ions which move relatively freely in the surrounding electrolyte.

For the low potential case, under this assumption, by using Equation (34), Equation (55) reduces to

(56) $$\phi_{i}\left(r\right)=r+\frac{a^{3}}{2r^{2}}\;$$

and

(57) $$G\left(r\right)=\frac{\varepsilon_{r}\varepsilon_{0}\kappa^{2}a\zeta }{\eta }\left(1+\frac{a^{3}}{2r^{3}}\right)\frac{1+\kappa r}{r^{2}}e^{-\kappa (r-a)}$$

By substituting Equations (56) and (57) into Equation (53), we obtain

$$\mu =\frac{2\varepsilon_{r}\varepsilon_{0}\kappa^{2}a\zeta }{3\eta \lambda^{2}\Omega }\int_{a}^{\infty }\left\{\left(1+\lambda r\right)e^{-\lambda \left(r-a\right)}-\left(1+\lambda a\right)-\frac{\lambda^{2}a^{2}}{3}\left(\frac{3\eta_{d}+3\eta +\eta \lambda a}{3\eta_{d}+2\eta }\right)\left(1-\frac{r^{3}}{a}\right)\right\}\left(1+\frac{a^{3}}{2r^{3}}\right)\frac{1+\kappa r}{r^{2}}e^{-\kappa (r-a)}dr$$

(58) $$-\frac{1+\lambda a}{\Omega \left(3\eta_{d}+2\eta \right)}\;\sum_{i=1}^{N}K_{i}z_{i}en_{i}^{\infty }$$

Using Equations (35) and (36), the second term on the right-hand side of Equation (58) can further be transformed into

(59) $$-\frac{1+\lambda a}{\Omega \left(3\eta_{d}+2\eta \right)}\;\sum_{i=1}^{N}K_{i}z_{i}en_{i}^{\infty }=-\frac{1+\lambda a}{\Omega \left(3\eta_{d}+2\eta \right)}\;\sigma^{\left(0\right)}=-\frac{\varepsilon_{r}\varepsilon_{0}\zeta \left(1+\lambda a\right)}{\Omega a\left(3\eta_{d}+2\eta \right)}\;(1+\kappa a)\;\;$$

By substituting Equation (59) into Equation (58), we finally obtain the following expression for the gel electrophoretic mobility *μ* of the oil drop:

$$\mu =\frac{2\varepsilon_{r}\varepsilon_{0}\kappa^{2}a\zeta }{3\eta \lambda^{2}\Omega }\int_{a}^{\infty }\left\{\left(1+\lambda r\right)e^{-\lambda \left(r-a\right)}-\left(1+\lambda a\right)-\frac{\lambda^{2}a^{2}}{3}\left(\frac{3\eta_{d}+3\eta +\eta \lambda a}{3\eta_{d}+2\eta }\right)\left(1-\frac{r^{3}}{a}\right)\right\}\left(1+\frac{a^{3}}{2r^{3}}\right)\frac{1+\kappa r}{r^{2}}e^{-\kappa (r-a)}dr$$

(60) $$-\frac{\varepsilon_{r}\varepsilon_{0}\zeta \left(1+\lambda a\right)}{\Omega a\left(3\eta_{d}+2\eta \right)}\;(1+\kappa a)$$

Equation (60) can be rewritten in terms of the exponential integrals, that is,

$$\mu =\frac{{2\varepsilon }_{r}\varepsilon_{o}\zeta }{3\eta \Omega }\left[\frac{3\eta_{d}+3\eta +\eta \lambda a}{3\eta_{d}+2\eta }+\frac{\kappa \lambda a}{\kappa +\lambda }-\frac{\eta (1+\lambda a)(3+\kappa a)}{2(3\eta_{d}+2\eta )}+\frac{3\kappa^{2}}{2\lambda^{2}}\left\{1+\lambda a+\frac{\lambda^{2}a^{2}}{3}\left(\frac{3\eta_{d}+3\eta +\eta \lambda a}{3\eta_{d}+2\eta }\right)\right\}e^{\kappa a}E_{5}\left(\kappa a\right)\right.$$

(61) $$-\frac{3\kappa^{2}}{2\lambda^{2}}e^{\left(\kappa +\lambda \right)a}\left.\left\{E_{5}\left(\left(\kappa +\lambda \right)a\right)+\lambda aE_{4}\left(\left(\kappa +\lambda \right)a\right)+\frac{\lambda^{2}a^{2}}{3}E_{3}\left(\left(\kappa +\lambda \right)a\right)\right\}\right]$$

where

(62) $$E_{n}\left(\kappa a\right)={\left(\kappa a\right)}^{n-1}\int_{\kappa a}^{\infty }\frac{e^{-\kappa t}}{t^{n}}dt$$

is the exponential integral of order *n*.

Equation (61) is the required approximate expression for the electrophoretic mobility *μ* of a weakly charged oil drop in an uncharged polymer gel medium.

Consider several limiting cases.

(i)
In the limit of *λa* → 0, Equation (61) reduces to
  (63) $$\mu =\frac{\varepsilon_{r}\varepsilon_{o}\zeta }{\eta }\left\{\frac{{3\eta }_{d}}{{3\eta }_{d}+2\eta }+2e^{\kappa a}E_{5}\left(\kappa a\right)-\left(\frac{15\eta_{d}}{{3\eta }_{d}+2\eta }\right)e^{\kappa a}E_{7}\left(\kappa a\right)\right\}$$
  which agrees with the electrophoretic mobility *μ* for the free-solution electrophoresis of a weakly charged oil drop [52]. In the further limit of *η*_d_/*η* → ∞, Equation (63) tends to
  (64) $$\mu =\frac{\varepsilon_{r}\varepsilon_{o}\zeta }{\eta }\left\{1+2e^{\kappa a}E_{5}\left(\kappa a\right)-5e^{\kappa a}E_{7}\left(\kappa a\right)\right\}$$
  which agrees with Henry’s electrophoretic mobility of a rigid sphere in a free-electrolyte solution [30].
(ii)
In the limit of *κa* → ∞ (Smoluchowski limit), Equation (61) becomes
  (65) $$\mu =\frac{\varepsilon_{r}\varepsilon_{o}\zeta (1+\lambda a)}{\eta \Omega }\left(\frac{{3\eta }_{d}}{{3\eta }_{d}+2\eta }\right)$$
(iii)
In the limit of *κa* → 0 (Hückel limit), Equation (61) becomes
  (66) $$\mu =\frac{\varepsilon_{r}\varepsilon_{o}\zeta }{3\eta \Omega }\left(\frac{6\eta_{d}+3\eta -\eta \lambda a}{3\eta_{d}+2\eta }\right)\;$$

Now, we define the scaled gel electrophoretic mobility *f*(*κa*, *λa*, *η*_d_/*η*) as

(67) $$\mu =\frac{\varepsilon_{r}\varepsilon_{o}\zeta }{\eta }f\left(\kappa a,\lambda a,\frac{\eta_{d}}{\eta }\right)$$

where

$$f\left(\kappa a,\lambda a,\frac{\eta_{d}}{\eta }\right)=\frac{2}{3\Omega }\left[\frac{3\eta_{d}+3\eta +\eta \lambda a}{3\eta_{d}+2\eta }\right.+\frac{\kappa \lambda a}{\kappa +\lambda }-\frac{\eta (1+\lambda a)(3+\kappa a)}{2(3\eta_{d}+2\eta )}$$

$$+\frac{3\kappa^{2}}{2\lambda^{2}}\left\{1+\lambda a+\frac{\lambda^{2}a^{2}}{3}\left(\frac{3\eta_{d}+3\eta +\eta \lambda a}{3\eta_{d}+2\eta }\right)\right\}e^{\kappa a}E_{5}\left(\kappa a\right)$$

(68) $$-\frac{3\kappa^{2}}{2\lambda^{2}}e^{\left(\kappa +\lambda \right)a}\left.\left\{E_{5}\left(\left(\kappa +\lambda \right)a\right)+\lambda aE_{4}\left(\left(\kappa +\lambda \right)a\right)+\frac{\lambda^{2}a^{2}}{3}E_{3}\left(\left(\kappa +\lambda \right)a\right)\right\}\right]$$

In the limit *λa* → 0 and *η*_d_/*η* → ∞, Equation (68) reduces to the following Henry function of a spherical rigid particle in a free-electrolyte solution [30]:

(69) $$f\left(\kappa a\right)=1+2e^{\kappa a}E_{5}\left(\kappa a\right)-5e^{\kappa a}E_{7}\left(\kappa a\right)$$

**Figure 2:**
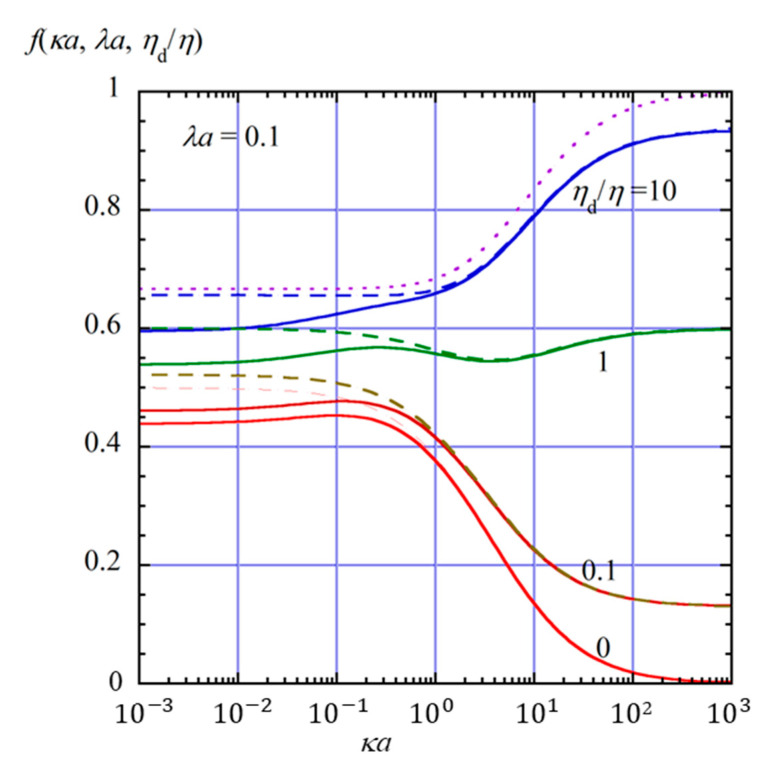
Scaled gel electrophoretic mobility *f*(*κa*, *λa*, *η*_d_/*η*) of an oil drop as a function of *κa* and *η*_d_/*η =* 2 at *λa* = 0.1 (solid lines). Dashed lines show the results for free-solution electrophoresis (*λa* = 0), and the dotted line represents the Henry function *f*(*κa*) for free-solution electrophoresis for a rigid sphere (*λa* = 0 and *η*_d_/*η* = ∞).

**Figure 3:**
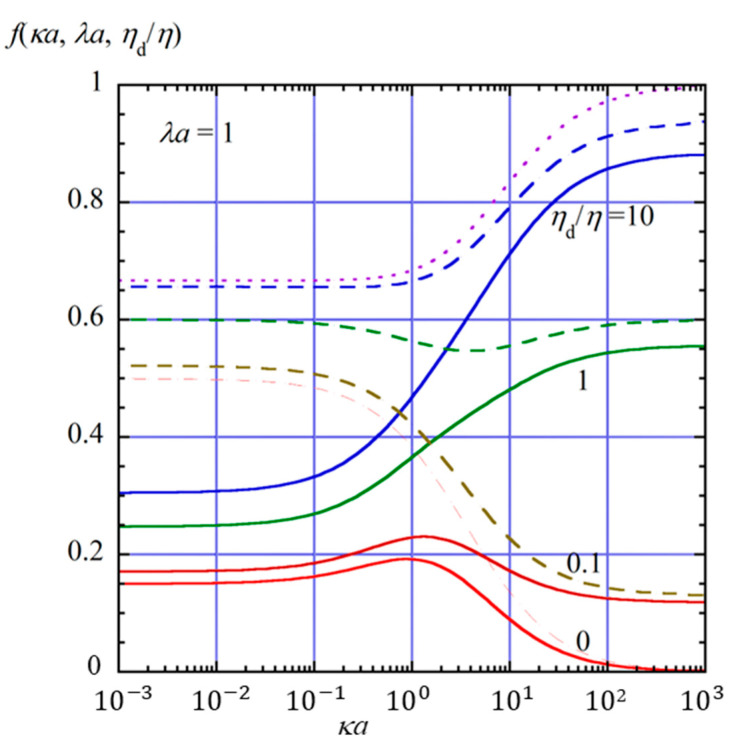
Same as Figure 2, but for *λa* = 1.

**Figure 4:**
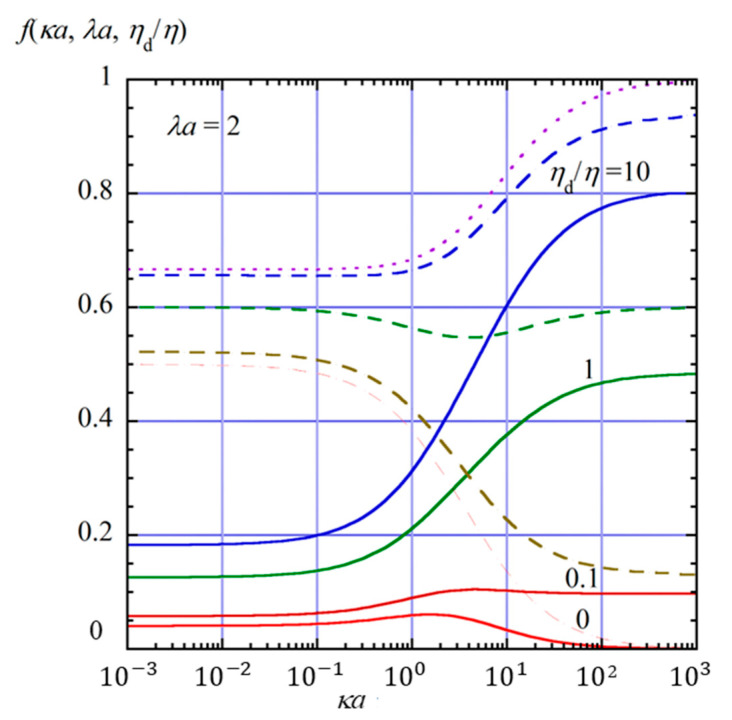
Same as Figure 2, but for *λa* = 2.

Figure 2, Figure 3 and Figure 4 show the scaled gel electrophoretic mobility *f*(*κa*, *λa*, *η*_d_/*η*) as a function of *κa* for four values of *η*_d_/*η* (0, 0.1, 1, 10) at *λa* = 0.1 (Figure 2), 1 (Figure 3), and 2 (Figure 4) indicated by solid lines. In these figures, for comparison, the results for the free-solution electrophoresis [52] are also given by dashed lines, and the result based on the Henry function (Equation (69) for the free-solution electrophoresis of a rigid sphere is shown by dotted lines.

As shown in these figures, *f*(*κa*, *λa*, *η*_d_/*η*) decreases as *λa* increase; that is, the hydrodynamic resistance from the surrounding gel becomes larger. This reduction in gel electrophoretic mobility results from the frictional drag exerted by the gel matrix, which opposes the motion of the drop under the applied electric field. In the opposite limit *λa* → 0, the present results approach those for the free-solution electrophoresis of an oil drop (Equation (63), shown by dashed lines), which further reduce to Henry’s mobility for a rigid sphere (Equation (64) or Equation (69), shown by dotted lines).

The scaled electrophoretic mobility *f*(*κa*, *λa*, *η*_d_/*η*) of the oil drop also decreases with decreasing internal viscosity *η*_d_. This behavior can be explained as follows. The electrophoretic mobility of an oil drop depends on the viscosity of the internal fluid. According to the theory developed by Baygents and Saville [40], the stress balance at the drop surface includes the hydrodynamic, Maxwell, and Marangoni stresses. When the viscosity of the internal fluid is low, the drop is more susceptible to interfacial flows driven by these stresses. In particular, the Marangoni and Maxwell stresses, which arise from the gradients of electric potential and ion concentration along the drop surface, tend to generate surface flows that oppose the electrophoretic motion. These flow-induced stresses are more effective at suppressing drop motion when the internal viscosity is small, since the internal fluid offers less resistance to interfacial shear. As a result, the electrophoretic mobility increases with increasing internal viscosity *η*_d_. In contrast, for weakly charged perfectly conducting drops such as mercury, the electrophoretic mobility decreases with increasing internal viscosity *η*_d_, exhibiting the opposite dependence on the internal viscosity *η*_d_ compared to oil drops. This is because the contributions of the Maxwell and Marangoni stresses are absent in the case of perfectly conducting drops.

Furthermore, the suppressive effects of the Maxwell and Marangoni stresses also depend strongly on the electrolyte concentration. As the salt concentration increases, the Debye length 1/*κ* decreases, resulting in sharper gradients of electric potential and ion concentration near the drop surface. These sharper interfacial gradients enhance both the Maxwell stress, which originates from discontinuities in the electric field, and the Marangoni stress, which results from surface tension gradients induced by ionic adsorption. Consequently, the interfacial stresses become more pronounced at higher salt concentrations, leading to stronger suppression of the electrophoretic motion. 

**Figure 5:**
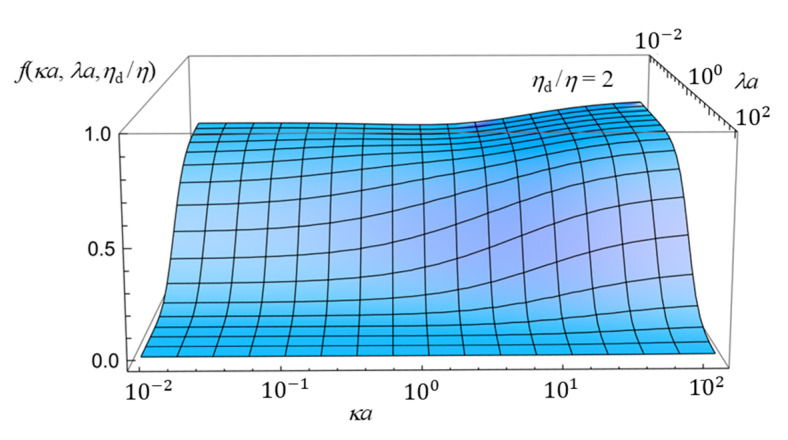
Three-dimensional plot of the scaled gel electrophoretic mobility *f*(*ka*, *la*, *h*_d_/*h*) of an oil drop as a function of *ka* and *la* at *h*_d_/*h* = 2.

**Figure 6:**
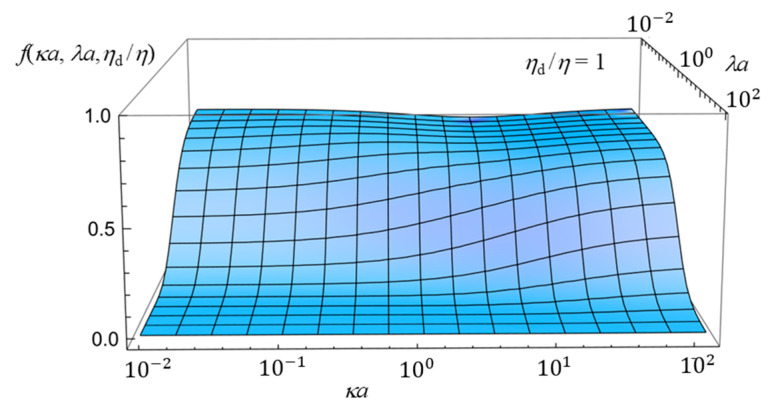
Same as Figure 5, but for *η*_d_/*η* = 1.

**Figure 7:**
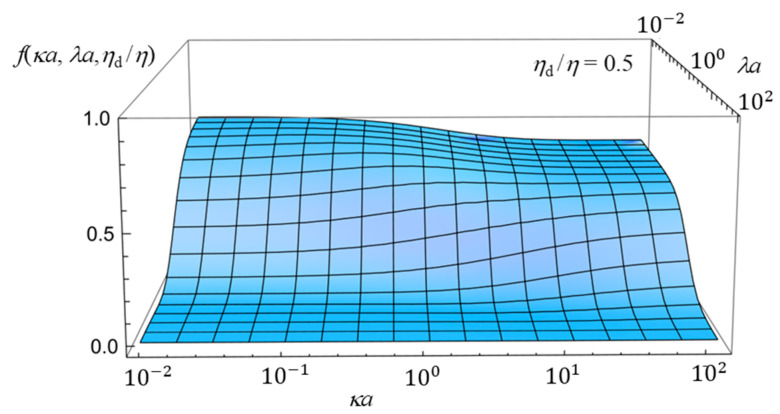
Same as Figure 5, but for *η*_d_/*η* = 0.5.

To further enhance the understanding of how the electrophoretic mobility depends on the combined effects of the three parameters—namely, the Debye length, the Brinkman screening length, and the viscosity of the oil drop—we now present three-dimensional plots in Figure 5, Figure 6 and Figure 7. Although the essential trends can already be captured by the two-dimensional plots (Figure 2, Figure 3 and Figure 4) discussed above, the three-dimensional representations provide a more comprehensive view of the parameter interdependencies and allow for clearer visualization of how mobility varies across broader parameter spaces. Figure 5, Figure 6 and Figure 7 show the three-dimensional plot of *f*(*ka*, *la*, *h*_d_/*h*) as a function of *ka* and *la* for three values of *η*_d_/*η*, showing how *f*(*κa*, *λa*, *η*_d_/*η*) depends on *κa*, *λa*, and *η*_d_/*η*.

The amount of adsorbed ions on the oil drop surface is proportional to the product of the bulk concentration and the adsorption constant *K_i_* (see Equation (14)), which in turn determines the surface charge density *σ* (Equation (15)). Under the low potential approximation, the zeta potential *ζ* is approximately proportional to the equilibrium surface charge density *σ*^(0)^ (see Equation (35)), and, thus, the electrophoretic mobility *μ* increases with increasing *Ki*. Accordingly, if the zeta potential *ζ* is experimentally known, the equilibrium surface charge density *σ*^(0)^ can be estimated using Equation (35), and then Equations (35) and (36) can be used to infer the value of *K_i_* for a given bulk ion concentration.

## 3. Conclusions

In this study, we have derived a simple closed-form analytical expression (Equation (61)) for the electrophoretic mobility *μ* of a weakly charged oil drop migrating through an uncharged polymer gel medium saturated with an aqueous electrolyte solution. The formulation is constructed under the assumption that the drop acquires its surface charge solely through the selective adsorption of ions from the surrounding electrolyte onto its surface. This model captures a more realistic charging mechanism than those assuming fixed surface potential or constant surface charge density.

The motion of the drop is driven primarily by long-range hydrodynamic effects from the gel matrix, which are described using the Debye–Bueche–Brinkman continuum model [36,37]. This framework accounts for the hydrodynamic resistance exerted by the polymer strands in the gel, modeled as a continuous distribution of frictional obstacles. The analysis builds on a simplified version of the Baygents–Saville theory [40], which neglects ion penetration into the drop interior and thereby focuses attention on surface processes that govern electrokinetic behavior.

A notable feature of the resulting mobility expression is that it explicitly incorporates the Marangoni effect arising from interfacial tension gradients. This effect plays an important role in the electrokinetics of liquid drops, as spatial variations in interfacial tension—driven by non-uniform ion adsorption—can significantly alter the flow field around the drop and hence affect its mobility. Our model accounts for this coupling and provides a more complete description of electrophoretic transport under weak-field conditions.

The analytical nature of the result enables straightforward evaluation of the dependence of drop mobility on key system parameters, such as the zeta potential, ionic adsorption constants, electrolyte composition, and gel permeability. As such, the present study offers a valuable theoretical framework for predicting and interpreting experimental data on drop transport in soft porous media. This is particularly relevant to emerging applications in microfluidics, drug delivery systems, and the design of bio-inspired materials, where liquid drops and gel-like environments are commonly encountered. For example, the present model may aid in predicting the electrophoretic motion of drops in microfluidic channels used for biochemical assays, estimating the transport of charged drug-loaded drops through hydrogel-based tissues, or optimizing the design of synthetic gels that emulate biological transport mechanisms.

Furthermore, the present formulation may serve as a foundation for extending the theory to more complex systems, such as drops with partial internal conductivity, non-uniform gel charge distributions, or time-dependent electric fields. While the present analysis focuses on spherical oil drops, the underlying framework may also be generalized to model the motion of soft particles, such as vesicles, polymeric microgels, or biological cells, by incorporating additional features such as deformability, non-uniform internal conductivity, or spatial variations in interfacial properties. For comprehensive reviews and foundational theories on the electrophoresis of soft particles, the reader is referred to Ashrafizadeh et al. [53].

The analytic expression derived in this study for the electrophoretic mobility of oil drops may be applicable to soft-matter systems, such as the electrophoretic transport of drug-loaded oil drops dispersed in hydrogels, biomimetic separation processes, and droplet-based microfluidic platforms, where control of drop motion under electric fields is essential (see, e.g., [54]).

In summary, the analytic expression obtained in this work advances the theoretical understanding of electrophoresis of oil drops in gel media by capturing key physicochemical effects—such as ion adsorption and Marangoni flow—in a tractable yet physically grounded framework. It contributes to the broader effort to bridge microscopic surface interactions and macroscopic transport behavior in complex soft-matter systems.

While direct comparison with experimental data is beyond the scope of the present theoretical study, the derived formula offers a practical and convenient tool for experimentalists. In particular, it provides a straightforward means to estimate the zeta potential from measured electrophoretic mobilities of liquid drops in gel environments, analogous to the well-established Henry’s formula for rigid particles. We anticipate that this work will stimulate and support future experimental investigations aimed at validating and applying these theoretical predictions.

## 4. Materials and Methods

This paper presents a theoretical analysis based on fundamental electrokinetic equations under weak-field conditions. No experimental or numerical simulation methods were employed. All derivations and results are described in Section 2.

## Data Availability

Data are contained within the article.
